# Autosomal dominant GDAP1 mutation with severe phenotype and respiratory involvement: A case report

**DOI:** 10.3389/fneur.2022.905725

**Published:** 2022-10-24

**Authors:** Adrian Rodriguez-Hernandez, Meagan Mayo, Lilibeth Jauregui, Pooja Patel

**Affiliations:** ^1^Department of Neurology, Charles E. Schmidt College of Medicine, Florida Atlantic University, Boca Raton, FL, United States; ^2^Department of Internal Medicine, Charles E. Schmidt College of Medicine, Florida Atlantic University, Boca Raton, FL, United States; ^3^Department of Neurology, Marcus Neuroscience Institute, Boca Raton, FL, United States

**Keywords:** Charcot Marie Tooth, GDAP1, case report, CMT2K, neuropathy

## Abstract

Charcot Marie Tooth (CMT) is a heterogeneous group of genetic disorders characterized by progressive motor and sensory neuropathy. CMT is a multi-gene disorder with several possible mutations responsible for a wide range of clinical presentations. A specific mutation of the ganglioside-induced-differentiation-associated protein 1 (*GDAP1*) gene is associated with the axonal subtype of CMT (CMT2K) which is inherited in an autosomal dominant fashion, as well as the demyelinating subtype (CMT4A) which is inherited in an autosomal recessive pattern. Phenotypic disease expression is largely dependent on these inheritance patterns. While the autosomal recessive form (CMT4A) exhibits severe disease with an early onset, the autosomal dominant variant (CMT2K) tends to have milder phenotypes and a later onset. We describe an atypical presentation of a patient with severe CMT2K with rapidly progressive polyneuropathy, respiratory failure, and dysphonia. We suggest that this case will inspire further evaluation of disease heterogeneity and variants.

## Introduction

Charcot Marie Tooth (CMT) is characterized by a clinically and genetically miscellaneous group of neuropathies, spanning a variety of Mendelian inheritance patterns. Greater than 1,000 different mutations in 80 disease-associated genes have been identified (1). Mutations in the ganglioside-induced-differentiation-associated protein 1 gene (*GDAP1*) are responsible for a subgroup of the hereditary motor and sensory neuropathies seen in Charcot Marie Tooth (2).

*GDAP1*-related mutations may be inherited in an autosomal recessive pattern (more common) or in an autosomal dominant pattern. *GDAP1* autosomal recessive inherited mutations, termed CMT4A, are characterized by demyelinating, axonal, or intermediate disease forms. CMT4A causes an early-onset, severe neuropathy that affects all extremities and frequently leads to wheelchair dependence in young adult life (second-third decade). In the advanced stages of the disease, most of these patients acquire vocal cord paresis (unilateral or bilateral) and diaphragmatic weakness. On the other hand, autosomal dominant inherited mutations, termed CMT2K, are characterized by axonal disease and tend to have a more benign presentation characterized by slow progression, adult-onset, and more distal involvement. Most of these patients remain ambulant throughout their lives (3, 4). In isolated patients, CMT2K has been associated with characteristics more commonly associated with CMT4A, such as dysphonia or dysautonomia (5).

This case report describes an atypical course of CMT2K with rapidly progressive polyneuropathy, dysphonia, and respiratory failure in a patient with adult-onset, autosomal dominant disease.

## Case presentation

A previously healthy and physically active 63-year-old male presented to the hospital due to a sensation of chest “heaviness” and dysphonia. Further history revealed that for the past 2 years, the patient had progressive paresthesias, cramping, and weakness beginning in the right lower extremity and then spreading to the left lower extremity and distal upper extremities bilaterally. He was now mostly confined to a wheelchair after being diagnosed with CMT 1 year ago.

**Figure 1:**
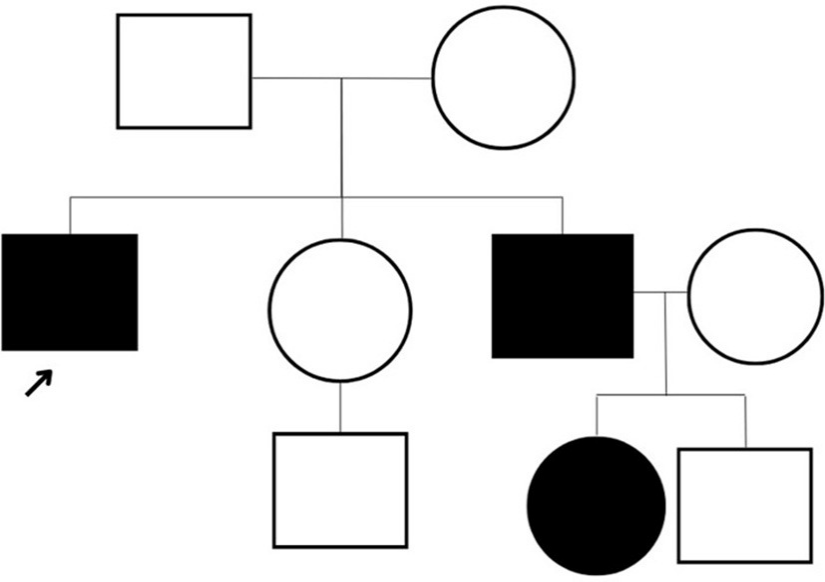
The three-generation pedigree of the family.

The patient underwent a comprehensive screen of 81 genes associated with peripheral neuropathies. Genomic deoxyribonucleic acid (gDNA) was isolated from the patient's whole blood. Sequence enrichment of the targeted coding exons and adjacent intronic nucleotides was carried out by a bait capture methodology using long biotinylated oligonucleotide probes followed by polymerase chain reaction (PCR) and Next-Generation sequencing. A compound heterozygous p. R120W mutation (also known as c.358C > T) was found at exon 3 of the *GDAP1* gene (NM_018972.4); resulting from a C to T substitution at nucleotide position 358. This mutation is described as “Pathogenic” (class 5) according to the ACMG guideline. A survey of the patient's family history also revealed an extensive incidence of CMT consistent with an autosomal dominant inheritance pattern. Disease was absent in both parents, with the patient's brother diagnosed at age five and the brother's daughter at age four. However, these family members presented with milder symptoms such as difficulty walking, arched feet, and wasting of the muscles in the lower extremities (Figure 1).

The patient's neurological examination revealed weakness of the bilateral upper extremities, bilateral intrinsic hand muscles, and right wrist extensors with compromised handwriting. Additionally, the patient had bilateral proximal leg weakness, including impaired foot flexion and dorsiflexion. There was generalized areflexia with reduced pinprick, vibration, and thermic sensation in all extremities (up to the knees and wrists). Based on these findings, the patient was assigned a CMT neuropathy scale of 24/36, which indicates severe neuropathy. In regard to the patient's initial concern of “chest heaviness”, chest X-ray was without abnormalities, although the patient was mildly tachypneic with the use of accessory muscles of respiration. The cardiac examination was unremarkable.

Laboratory tests did not reveal any acute abnormalities. Infections and acute coronary syndrome were excluded. Notably, there was an absence of paraprotein and a normal creatinine kinase. Anti-Ach binding, anti-Ach blocking, antineuronal, antiganglioside, and anti-myelin-associated glycoprotein precursor antibodies were all negative. The patient refused lumbar puncture for cerebrospinal fluid (CSF) studies (he verbally explained normal CSF results from 1 year ago, although we were unable to obtain this report). MRI of the brain and cervical spine, as well CT of the chest, were without abnormalities.

On further workup, nerve conduction studies (NCS) demonstrated evidence of right median axonopathy, left median and right ulnar mixed type neuropathy, and demyelinating left ulnar neuropathy. There was also evidence of a severe generalized sensory and motor neuropathy of the lower extremities with an absence of F and H waves (Table 1). Due to the patient's decreased phonation and weak cough, intrahospital speech therapy suggested outpatient follow up for formal evaluation of probable vocal cord impairment.

**Table 1:** Motor and sensory nerve conduction studies of the bilateral upper and lower extremities.

**Nerve**	**Stimulation site**	**Distance (cm)**	**Recording site**	**Latency (ms)**			**Amplitude (CMAP Mv; SNAP uV)**			**Velocity (m/s)**		
**Motor**				**R**	**L**	**NL**	**R**	**L**	**NL**	**R**	**L**	**NL**
Median	Wrist	22.5	APB	5.2	5	<4.2	0.3	0.8	>5	52	47	>50
Median	Elbow		APB	9.5	9.8		0.3	0.9				
Ulnar	Wrist	22	ADM	4.5	3.4	<4.2	2	4.3	>5	50	49	>53
Ulnar	B elbow	10	ADM	8.9	7.9		2	3.4		59	53	>53
Ulnar	A Elbow		ADM	10.6	9.8		2.4	3.9				
Peroneal	Fib Head		EDB	*NR	*NR	<6.1	*NR	*NR	>2.5	*NR	*NR	>40
Peroneal	Pop fossa	10	EDB	*NR	*NR		*NR	*NR		*NR	*NR	
Tibial	Ankle		AHB	*NR	*NR	<6.1	*NR	*NR	>3.0	*NR	*NR	>35
Tibial	Pop fossa		AHB	*NR	*NR		*NR	*NR		*NR	*NR	
**Sensory**
Median	Wrist	14	Dig II	4	3.6	<3.6	38.4	28.5	>10	35	39	>39
Ulnar	Wrist	14	Dig V	4.1	3.1	<3.7	11.2	22.9	>15	34	45	>38
Radial	Forearm		Wrist	2.2	2	<3.1	26.5	24.4	>15			
Sural	Calf		Ankle	4.4	4.6	<4.0	6.1	4.7	>5.0	32	30	>35

ADM, abductor digiti minimi; AHB, abductor hallucis brevis; APB, abductor pollicis brevis; cm, centimeters; Dig, digit; EDB, extensor digitorum brevis; Fib, fibular; L, left; m/s, meters per second; ms, millisecond; NL, normal limit; Pop, popliteal; R, right; SNAP, sensory nerve action; uV, microvolt; NR, no response.

With imminent threat to life ruled out, the patient was ultimately discharged. Unfortunately, he was lost to follow up and returned to the hospital 3 weeks later with worsening respiratory failure and dysphonia. At this time, arterial blood gas revealed persistent hypercapnia with hypoxemia, requiring Bilevel Positive Airway Pressure (BIPAP). Chest X-ray showed mild elevation of the left hemidiaphragm. An extensive workup for respiratory insufficiency excluded electrolyte abnormalities, myasthenia, autoimmune disease, substance abuse, infection, pulmonary embolism, spinal cord injury, and structural brain abnormalities. The patient developed worsening mental status in the setting of refractory hypercapnia and respiratory muscle fatigue. Per his wishes, the patient was not intubated and ultimately was transitioned to hospice care within 6 weeks of his initial presentation to this hospital (Table 2).

**Table 2:** Chronological clinical course of the patient.

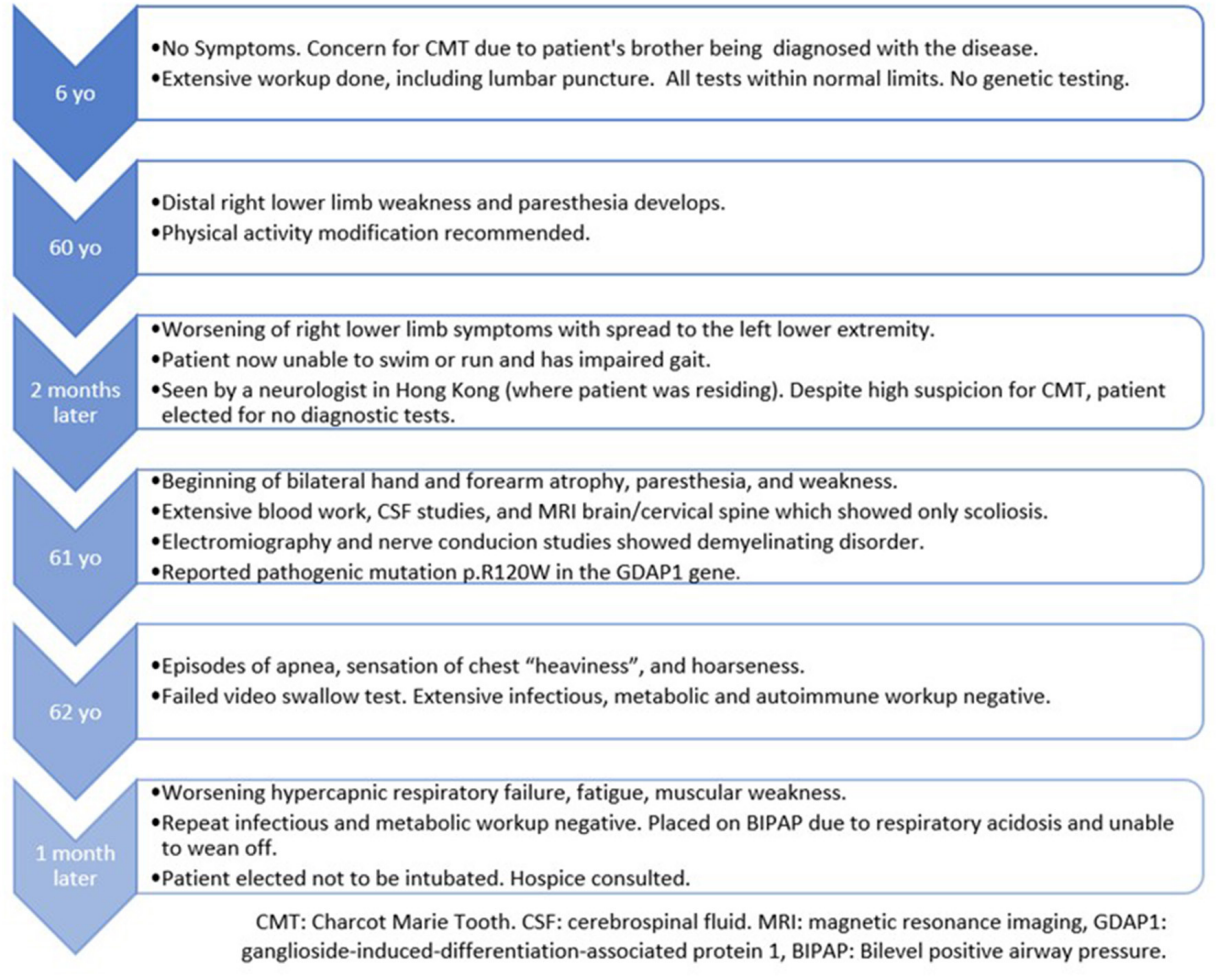

## Discussion

Charcot-Marie Tooth presents heterogeneously with progressive motor and sensory polyneuropathy leading to weakness, muscle atrophy, loss of sensation, loss of deep tendon reflexes, and foot deformities. Mutations in the *GDAP1* gene are responsible for both an autosomal recessive early-onset severe demyelinating form of CMT (CMT4A) and a late-onset dominant form (CMT2K)—such as was seen in our patient. Specifically, our patient possessed a p.R120w mutation located in coding exon 3 in the *GDAP1* gene. This pathogenic mutation is the most common variant seen within CMT2K (3, 8). However, the severity of our patient's clinical presentation was inconsistent with current knowledge of this CMT2K subtype (Table 3).

**Table 3:** Clinical characteristics of patients with various autosomal dominant mutations in the ganglioside induced differentiation associated protein 1 (GDAP1) gene.

**Study**	**Kim et al. (6)**	**Pakhrin et al. (7)**	**Sivera et al. (8)**	**Fu et al. (9)**	**Pezzini et al. (5)**	**Auranen et al. (10)**	**Chung et al. (11)**
Country	Korea	China	Spain	China	Italy	Finland	Korea
Number of Patients	7	9	58	3	26	14	2
Mutation(s)	R120W	V219G	R120W	R120W	R120W	H123R	Q218E
	Q218E	Q38R	R22GK	C240Y	R226S	V795I (MFN2)	
	R226K		T157P		Q218E		
					H123R		
					Arg120Gly		
Age onset (mean)	19.7 yrs	15.8 yrs	23.8 yrs	3 yrs	19.8 yrs	33.5 yrs	24.5 yrs
FDS Score (mean)	2.2	NA	1.3	NA	NA	NA	NA
CMTNS (mean/range)	11.1/6–25	NA/3–13	7.3/0–26	12.5/12–13	NA	NA	NA
Muscle Weakness	All LL weakness (R22K > R120W)	Mild in LL	Distal LL	Distal and proximal	Distal LL	Distal > proximal	Moderate in distal LL and UL
	Hand weakness (in 5/7 patients)	Severe in hands (Q38R mutation only)		LL > UL	Distal UL (in 4/26 patients)		
Wheelchair bound (number of patients)	No	Yes (1)	No	Yes (1)	No	NA	No
Foot deformities (percentage of patients)	Yes	Yes	Yes (85.2%)	Yes (80%)	Yes (< 50%)	Yes	Yes
Sensory loss	V = P (28%)	Motor only	Non-specific sensory (57.1%)	V (33%)	NA	Motor only	Distal V and P
	V > P (58%)						
	Normal (14%)						
Vocal cord paresis diaphragmatic weakness hoarseness	No	No	No	No	No	Yes (1). Dysphonia (in MFN2 mutation)	No
Electrophysiological studies	CMAP: normal (UL), decreased (LL)	MNCV: 40.9 m/s	CMAP: decreased	MNCV/CMAP in 2 patients (mean): 56.1 m/s/2.1 mv	CMAP: Decreased (LL > UL)	SNAP: Decreased (LL > UL)	MNCV: mildly reduced/normal
	MNCV: decreased (LL) SNAP: decreased (LL > UL)	CMAP 2.1 mV	SNAP: decreased			MNCV: normal MNCV: normal	CMAP: mildly reduced/normal SNCV: normal.

CMTNS, charcot marie tooth examination score; FDS, functional disability score; LL, lower limbs; UL, upper limbs; V, vibration; P, pain; NA, not available; yrs, years; CMAP, compound muscle action potential; MNCV, motor nerve conduction velocity; SNAP, sensory nerve action potential; SNCV, sensory nerve conduction velocity; m/s, meter/second; mV, millivolts.

Although the clinical phenotype within CMT genotypes may differ between individuals in one family, this patient's rapidly progressive disease in his 6th decade of life is a unique presentation of CMT2K. As described by Silveira et al. (8), the CMT2K usually does not present with respiratory failure, stridor, or vocal cord dysfunction. Rather, these are common characteristics of recessive forms (CMT4A). Therefore, extensive workup to rule out neuromuscular, infectious, toxic, cardiac, and metabolic etiologies of respiratory failure is essential in such atypical presentations. Although Abbousonan et al. (12) described multiple disorders of pulmonary function, sleep, and upper airway in patients with other CMT variants, these have not been documented in the CMT2K form. We believe our patient had a rapidly progressive restrictive pulmonary impairment secondary to diaphragmatic nerve dysfunction, as well as vocal cord impairment secondary to peripheral neuropathy affecting the vagus nerve and its laryngeal branches.

CMT2K is responsible for axonal neuropathy that is associated with (relatively) normal nerve conduction velocities (NCV), loss of myelinated axons, and regenerative sprouting. On the other hand, CMT4A causes demyelinating, axonal, or mixed type CMT that is characterized by segmental demyelination with a diffuse reduction in NCV (13). In additional to an unusually rapid onset and severe disease course, our patient also had an atypical neuropathy consisting of a mainly mixed pattern with a probable right concomitant carpal tunnel syndrome.

To our knowledge, we present the only documented case of CMT2K with adult-onset rapidly progressive respiratory failure and acute vocal cord dysfunction with evidence of mixed demyelinating and axonal neuropathy. This suggests that the phenotypic presentations of various types of CMT may extend beyond our current genotypic cognizance. This case will hopefully serve to motivate further research, disease understanding, and treatment optimization for those diagnosed with Charcot Marie Tooth.

## Data availability statement

The original contributions presented in the study are included in the article/supplementary material, further inquiries can be directed to the corresponding author.

## Ethics statement

Written informed consent was obtained from the individual(s) for the publication of any potentially identifiable images or data included in this article.

## Author contributions

AR-H, MM, LJ, and PP contributed to the writing of the manuscript and critical revision of the manuscript. All authors gave important contributions to the final form of the manuscript.

## Conflict of interest

The authors declare that the research was conducted in the absence of any commercial or financial relationships that could be construed as a potential conflict of interest.

## Publisher's note

All claims expressed in this article are solely those of the authors and do not necessarily represent those of their affiliated organizations, or those of the publisher, the editors and the reviewers. Any product that may be evaluated in this article, or claim that may be made by its manufacturer, is not guaranteed or endorsed by the publisher.

## References

[1] Timmerman V Strickland AV Züchner S. Genetics of Charcot-Marie-Tooth (CMT) disease within the frame of the human genome project success. Genes. (2014) 5:13–32. 10.3390/genes5010013 24705285 PMC3978509

[2] Cuesta A Pedrola L Sevilla T García-Planells J Chumillas MJ Mayordomo F. The gene encoding ganglioside-induced differentiation-associated protein 1 is mutated in axonal Charcot-Marie-Tooth type 4A disease. Nat Genet. (2002) 30:22–5. 10.1038/ng798 11743580

[3] Zimoń M Baets J Fabrizi GM Jaakkola E Kabzińska D Pilch J. Dominant GDAP1 mutations cause predominantly mild CMT phenotypes. Neurology. (2011) 77:540–8. 10.1212/WNL.0b013e318228fc70 21753178 PMC3272385

[4] Sevilla T Jaijo T Nauffal D Collado D Chumillas MJ Vilchez JJ. Vocal cord paresis and diaphragmatic dysfunction are severe and frequent symptoms of GDAP1-associated neuropathy. Brain. (2008) 131(Pt 11):3051–61. 10.1093/brain/awn228 18812441

[5] Pezzini I Geroldi A Capponi S Gulli R Schenone A Grandis M. GDAP1 mutations in Italian axonal Charcot-Marie-Tooth patients: Phenotypic features and clinical course. Neuromuscul Disord. (2016) 26:26–32. 10.1016/j.nmd.2015.09.008 26525999

[6] Kim HS Kim HJ Nam SH Kim SB Choi YJ Lee KS. Clinical and neuroimaging features in Charcot-Marie-Tooth patients with GDAP1 mutations. J Clin Neurol. (2021) 17:52–62. 10.3988/jcn.2021.17.1.52 33480199 PMC7840330

[7] Pakhrin PS Xie Y Hu Z Li X Liu L Huang S. Genotype-phenotype correlation and frequency of distribution in a cohort of Chinese Charcot-Marie-Tooth patients associated with GDAP1 mutations. J Neurol. (2018) 265:637–46. 10.1007/s00415-018-8743-9 29372391

[8] Sivera R Frasquet M Lupo V García-Sobrino T Blanco-Arias P Pardo J. Distribution and genotype-phenotype correlation of GDAP1 mutations in Spain. Sci Rep. (2017) 7:6677. 10.1038/s41598-017-06894-6 28751717 PMC5532232

[9] Fu J Dai S Lu Y Wu R Wang Z Yuan Y. Similar clinical, pathological, and genetic features in Chinese patients with autosomal recessive and dominant Charcot-Marie-Tooth disease type 2K. Neuromuscul Disord. (2017) 27:760–5. 10.1016/j.nmd.2017.04.001 28495047

[10] Auranen M Ylikallio E Toppila J Somer M Kiuru-Enari S Tyynismaa H. Dominant GDAP1 founder mutation is a common cause of axonal Charcot-Marie-Tooth disease in Finland. Neurogenetics. (2013) 14:123–32. 10.1007/s10048-013-0358-9 23456260

[11] Chung KW Kim SM Sunwoo IN Cho SY Hwang SJ Kim J. A novel GDAP1 Q218E mutation in autosomal dominant Charcot-Marie-Tooth disease. J Hum Genet. (2008) 53:360–4. 10.1007/s10038-008-0249-3 18231710

[12] Aboussouan LS Lewis RA Shy ME. Disorders of pulmonary function, sleep, and the upper airway in Charcot-Marie-Tooth disease. Lung. (2007) 185:1–7. 10.1007/s00408-006-0053-9 17294338

[13] Shy ME. Charcot-Marie-Tooth disease: an update. Curr Opin Neurol. (2004) 17:579–85. 10.1097/00019052-200410000-00008 15367862

